# Temporal trends in the discovery of human viruses

**DOI:** 10.1098/rspb.2008.0294

**Published:** 2008-05-27

**Authors:** Mark E.J Woolhouse, Richard Howey, Eleanor Gaunt, Liam Reilly, Margo Chase-Topping, Nick Savill

**Affiliations:** Centre for Infectious Diseases, Ashworth Laboratories, Kings Buildings, University of EdinburghEdinburgh EH9 3JT, UK

**Keywords:** discovery curve, emerging infectious diseases, public health, surveillance, virus species

## Abstract

On average, more than two new species of human virus are reported every year. We constructed the cumulative species discovery curve for human viruses going back to 1901. We fitted a statistical model to these data; the shape of the curve strongly suggests that the process of virus discovery is far from complete. We generated a 95% credible interval for the pool of as yet undiscovered virus species of 38–562. We extrapolated the curve and generated an estimate of 10–40 new species to be discovered by 2020. Although we cannot predict the level of health threat that these new viruses will present, we conclude that novel virus species must be anticipated in public health planning. More systematic virus discovery programmes, covering both humans and potential animal reservoirs of human viruses, should be considered.

## 1. Introduction

Despite long-standing interest in global biodiversity (May 1988), only recently has the diversity of human pathogens been catalogued (Taylor *et al.* 2001). Approximately 1400 pathogen species are currently recognized (Woolhouse & Gaunt 2007). Fewer than 200 of these are viruses, but novel virus species are being reported in humans at a rate of over two per year, much faster than for other kinds of pathogen (Woolhouse & Gaunt 2007). Novel viruses are a major public health concern, whether causing disease on the massive scale of HIV/AIDS, more transient events such as the SARS epidemic or potential future threats such as pandemic influenza. An analysis of temporal patterns of virus discovery is therefore of considerable interest.

Our analysis is based on the rate of accumulation of new human virus species: the ‘discovery curve’. Discovery curves have previously been used to estimate the total diversity of various plant and animal taxa (Dove & Cribb 2006; Bebber *et al.* 2007). However, to our knowledge, the discovery curves have not previously been compiled for any category of human pathogen. Having compiled the discovery curve, we proceed to develop a simple statistical model which we use to estimate the size of the pool of human virus species, *N*, and the expected rate of discovery of new species to 2020.

## 2. Material and methods

A standard method for estimating numbers of species is to extrapolate the cumulative species discovery curve (Bebber *et al.* 2007). We gathered data for this curve by systematically searching the primary literature for first reports of human infection with each of the currently recognized virus species, using species as defined by the International Committee on Taxonomy of Viruses (ICTV; http://www.ictvonline.org/). We note that the set of viruses we are interested in—those that can infect humans—is a small subset of the total (over 1500 species according to ICTV) and, as is discussed below, not a closed set because many of these viruses can also infect other hosts (Taylor *et al.* 2001). We regard this as analogous to constructing species discovery curves for any subdivision of geographical range or habitat. As we demonstrate below, this approach yields an excellent description of the discovery curve.

We used piecewise linear regression to test for changes in the slope of the discovery curve. The results suggested upswings in 1930 (95% CI, 1929–1933) and 1954 (1953–1956). We therefore restricted detailed analysis to the period 1954–2006.

We modelled discovery since 1954 assuming a total number of species available to be discovered (the species pool) of *N* virus species, each discovered in any given year with probability *p*. The model was fitted to the data and assessed using Markov chain Monte Carlo (MCMC)-based Bayesian inference, generating distributions and credible intervals for the parameters. The model defines the expected number of discovered viruses in year *t* as

(2.1) $$\lambda_{t}(N,p)=Np{\left(1-p\right)}^{t-1},$$

where year *t*=1 corresponds to 1954.

The binomial distribution *B*(*N*, *p*) can be accurately approximated by a Poisson distribution with parameter *Np* for the range of values of *N* and *p* of interest. We considered fitting a distribution for values of *p*; however, provided individual *p*-values are low there is minimal improvement in model fit. Thus, for a set of model parameters, the likelihood of observing data, *X*={*x*_*i*_}, the number of viruses discovered for years 1 to *k*, is given by

(2.2) $$L(X|N,p)=\prod_{i=1}^{k}\frac{\text{exp}(-\lambda_{i}(N,p))\lambda_{i}^{x_{i}}(N,p)}{x_{i}!}.$$

Parameter distributions for *N* and *p* were calculated using MCMC simulation using a standard Metropolis algorithm with flat prior information. It was necessary to compute a correlation matrix to define a joint proposal since *N* and *p* are closely correlated. We monitored convergence using two chains. Once they had converged, we had a burn in period of 10^5^ samples.

We compared the model with the observed data by calculating the mean, trend in the mean and variance for the number of virus species discovered per year (based on five million simulations using best-fit parameter values). The model was extrapolated to year 2020 by calculating the expected number of viruses discovered using the best-fit model. The 95% posterior prediction intervals were calculated using two million model simulations taking into account parameter uncertainty (as given by data from 1954 to 2006) and natural model simulation stochasticity.

As a validation exercise, the model was also fitted to the curve for accumulated virus families from 1954 using the same methods, except that the Poisson approximation no longer holds, so a binomial distribution was used. A family (based on current ICTV classifications) was added to the total when the first post-1954 species was allocated to that family. We tested the assumption that species can be randomly assigned to families (weighted by the size of the families) by noting the number of years in which 0, 1, 2, etc. virus families were discovered. This was done one million times to obtain a distribution for comparison with the observed values.

## 3. Results

From a comprehensive search of the primary literature, we found 188 virus species that have been reported to infect humans, going back to yellow fever virus in 1901 (table 1). Since then, the number of human virus species discovered in any given year has ranged from zero to six. As is typical (Bebber *et al.* 2007), the cumulative species discovery curve increases slowly initially and then more rapidly (figure 1). Piecewise linear regression suggests no further upswings since 1954, roughly corresponding to the advent of tissue culture techniques for virus detection (figure 1).

We confirmed that our model reproduced the observed slight downward trend in the rate of discovery since 1954 (figure 1) and the observed variance in the data from 1954 to 2006 (figure 2). The distribution of the number of virus species discovered per year shows slight overdispersion (mean=2.69; variance=3.07; variance-to-mean ratio greater than 1) which falls within the predicted range (mean=2.70 with 95% credible interval 2.41–3.00; variance=3.03 with interval 1.99–4.49). Together, these results support our choice of model, even though we do not explicitly consider heterogeneity in the probability of discovering a given species in any one year (*p*) or temporal variation in sampling effort, detection techniques and reporting.

Noting that *p* and *N* are highly correlated (figure 3), our best estimate for *p* is 0.015 (95% credible interval, 0.004–0.026) with 117 (38–562) so far undiscovered virus species. Extrapolating the discovery curve, allowing for parameter uncertainty and stochastic discovery, we obtain a best estimate of 22 new species (10–40) by 2020 (figure 1).

Data on the cumulative discovery of new virus families are also reproducible (figure 4). The predicted distribution of the number of virus families discovered per year (assuming random allocation of species to families) compares favourably with the observed distribution (figure 5). This provides further support for the appropriateness of our model.

## 4. Discussion

We conclude that it is extremely probable that new human viruses will continue to be discovered in the immediate future; we are not yet close to the end of the virus discovery curve. As a direct result of this, it is not possible to estimate the size of the species pool for human viruses with precision. However, in contrast to the negative assessment by Bebber *et al.* (2007) of the use of incomplete species accumulation curves, we consider that the upper and lower limits to our estimate of the size of the species pool are of interest and also have practical implications.

Current trends are consistent with a pool of at least 38 undiscovered species that will be reported at an average rate of at least approximately one per year to 2020. In this context, it is worth noting that three new species were reported in 2007: two polyoma viruses, Ki and Wu, and a reovirus, Melaka (Allander *et al.* 2007; Chua *et al.* 2007; Gaynor *et al.* 2007). Other viruses may have been reported but not yet classified. In practice, future rates of discovery will, of course, be affected by any major advances in virus detection technology or by any major shifts (upwards or downwards) in the effort expended on virus discovery programmes. Tissue culture was regarded as the ‘gold standard’ for virus detection up until a few years ago when molecular methods came to the fore (Storch 2007), although there has not been a detectable increase in discovery rates as a result. Indeed, it is striking that there have been no dramatic changes in the pattern of virus discovery for over 50 years; extrapolations from our data should therefore provide a useful benchmark for probable future discovery rates.

The upper limit for *N* is finite but large; we cannot rule out hundreds of novel human viruses to be reported in the future. There are two (not mutually exclusive) possible explanations for such a high level of diversity. First, it could reflect the largely unknown extant diversity of viruses in the non-human animal reservoirs that constitute the major source of emerging human pathogens (Taylor *et al.* 2001; Woolhouse & Gaunt 2007). The majority of human viruses are known to be capable of infecting non-human hosts (almost exclusively mammals and birds), and the animal origin of many apparently novel human viruses (e.g. HIV1 and HIV2, SARS CoV, Nipah virus) has been frequently remarked upon (Morse 1995; Woolhouse & Gowtage-Sequeria 2005; Wolfe *et al.* 2007); indeed, recently discovered viruses are even more likely to be associated with a non-human reservoir (Woolhouse & Gaunt 2007). All these observations are consistent with the idea that a significant fraction of viruses discovered in the last few decades is ecological ‘spillover’ from animal populations rather than newly evolved specialist human viruses. We have very limited knowledge of the diversity of viruses present in most mammal and bird species (with most attention having been paid to viruses of domestic animals; Cleaveland *et al.* 2001), so it is unclear for how long this process might continue.

An alternative explanation for a large pool of human viruses is that this reflects a high rate of evolution (within a reservoir population) of truly novel species capable of infecting humans. This hypothesis is difficult to test directly without much more comprehensive sequence data from both human and non-human virus populations. We note that the finite upper limit for the current estimate of *N* does not necessarily imply that the process of virus discovery is not open-ended (as a result of the evolution of new species) since there could be a low background rate of virus evolution, which will remain once extant diversity has been fully revealed. The balance between revealing extant diversity and the continual evolution of new species could be explored using a more complex model than equation (2.1); however, the available data are insufficient to yield useful estimates of the additional parameters required.

Although we cannot know in advance how big a threat they will pose, novel human viruses must be anticipated in public health planning and surveillance programmes for emerging infectious diseases (King *et al.* 2006; Jones *et al.* 2008). However, current approaches to virus discovery are largely passive, usually relying on investigation of reports of human disease with unfamiliar clinical symptoms and uncertain aetiology. Recently, there have been calls for more active discovery programmes for viruses and other pathogens involving ‘systematic sampling and phylogeographic analysis of related pathogens in diverse animal species’ (Wolfe *et al.* 2007). We consider that such calls are supported by the results reported here.

## Figures and Tables

**Figure 1:**
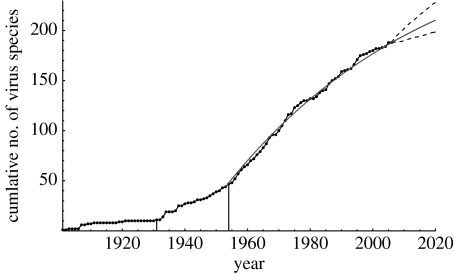
The discovery curve for human virus species. Cumulative number of species reported to infect humans (black circles and line). Statistically significant upward breakpoints are shown (vertical lines). Best-fit curve (solid line) and lower and upper 95% posterior prediction intervals (dashed lines) for extrapolation to 2020.

**Figure 2:**
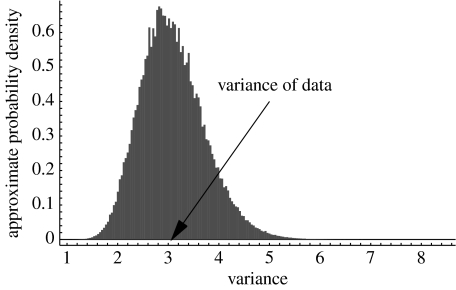
Approximate probability density of variance in simulated data from 1954 to 2006 for the best-fit model. Arrow shows observed value.

**Figure 3:**
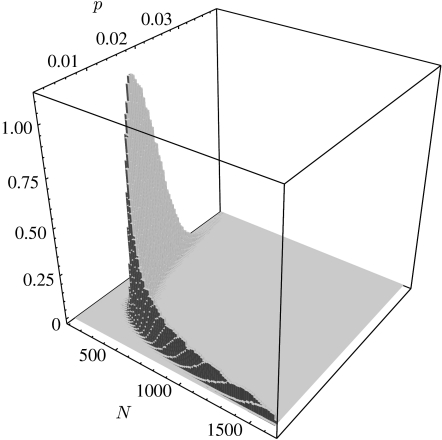
Approximate probability density function of parameter *p* and *N* generated by MCMC methods (see main text for details).

**Figure 4:**
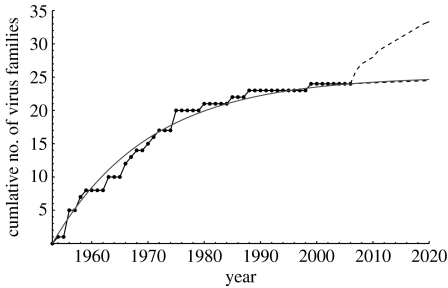
Accumulation of virus families associated with species discovered after 1954 (black circles and line). Best-fit curve (solid line) and lower and upper 95% posterior prediction intervals (dashed lines) extrapolated to 2020. Fitted parameter values are *N*=25 (95% credible intervals 24–37) and *p*=0.056 (0.027–0.089).

**Figure 5:**
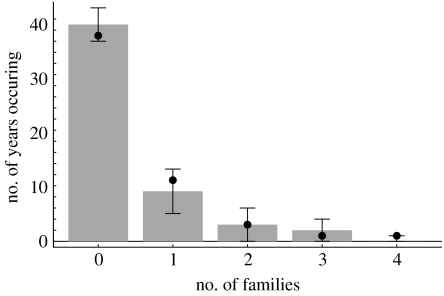
Frequency distribution for the number per year of virus families associated with species discovered from 1954 to 2006, generated by reassigning the discovered viruses to families, repeated 10^6^ times. Expected number with 95% credible intervals (bars) and data (black circles).

**Table 1:** List of viruses ordered by year of first report^a^ of human infection.

year	species	family
1901	Yellow fever virus	flavi
1903	Rabies virus	rhabdo
1907	Dengue virus	flavi
1907	Human papillomavirus	papilloma
1907	Molluscum contagiosum virus	pox
1907	Variola virus	pox
1909	Poliovirus	picorna
1911	Measles virus	paramyxo
1919	Human herpesvirus 3	herpes
1921	Human herpesvirus 1	herpes
1931	Rift Valley fever virus	bunya
1933	Influenza A virus	orthomyxo
1933	Lymphocytic choriomeningitis virus	arena
1933	St Louis encephalitis virus	flavi
1934	Cercopithecine herpes virus 1	herpes
1934	Japanese encephalitis virus	flavi
1934	Louping ill virus	flavi
1934	Mumps virus	paramyxo
1934	Orf virus	pox
1937	Tick-borne encephalitis virus	flavi
1938	Cowpox virus	pox
1938	Eastern equine encephalitis virus	toga
1938	Rubella virus	toga
1938	Venezuelan equine encephalitis virus	toga
1938	Western equine encephalitis virus	toga
1940	Influenza B virus	orthomyxo
1940	West Nile virus	flavi
1941	Bwamba virus	bunya
1943	Newcastle disease virus	paramyxo
1944	Sandfly fever Naples virus	bunya
1944	Sandfly fever Sicilian virus	bunya
1946	Colorado tick fever virus	reo
1947	Omsk haemorrhagic fever virus	flavi
1948	Encephalomyocarditis virus	picorna
1948	Human enterovirus C	picorna
1949	Human enterovirus A	picorna
1949	Human enterovirus B	picorna
1950	Influenza C virus	orthomyxo
1950	Vesicular stomatitis virus	rhabdo
1951	Bunyamwera virus	bunya
1952	California encephalitis virus	bunya
1952	Murray Valley encephalitis virus	flavi
1952	Ntaya virus	flavi
1953	Human rhinovirus A	picorna
1954	Human adenovirus B	adeno
1954	Human adenovirus C	adeno
1954	Human adenovirus E	adeno
1955	Human adenovirus D	adeno
1956	Chikungunya virus	toga
1956	Human herpesvirus 5	herpes
1956	Human parainfluenza virus 2	paramyxo
1956	Ilheus virus	flavi
1957	Human adenovirus A	adeno
1957	Human respiratory syncytial virus	paramyxo
1957	Kyasanur forest disease virus	flavi
1957	Mayaro virus	toga
1957	Wesselsbron virus	flavi
1958	Human parainfluenza virus 1	paramyxo
1958	Human parainfluenza virus 3	paramyxo
1958	Human parechovirus	picorna
1958	Junin virus	arena
1959	Banzi virus	flavi
1959	Guaroa virus	bunya
1959	Powassan virus	flavi
1960	Human parainfluenza virus 4	paramyxo
1960	Human rhinovirus B	picorna
1961	Caraparu virus	bunya
1961	Catu virus	bunya
1961	O'nyong-nyong virus	toga
1961	Oropouche virus	bunya
1962	Rio Bravo virus	flavi
1962	Sindbis virus	toga
1963	Equine rhinitis virus A	picorna
1963	Great Island virus	reo
1963	Pseudocowpox virus	pox
1963	Yaba monkey tumour virus	pox
1964	Human herpesvirus 4	herpes
1964	Machupo virus	arena
1964	Zika virus	flavi
1965	Chagres virus	bunya
1965	Foot and mouth disease virus	picorna
1965	Tanapox virus	pox
1965	Wyeomyia virus	bunya
1966	Changuinola virus	reo
1966	Human coronavirus 229E	corona
1966	Quaranfil virus	unassigned
1966	Saimiriine herpesvirus 1	herpes
1967	Chandipura virus	rhabdo
1967	Crimean-Congo haemorrhagic fever virus	bunya
1967	Human coronavirus OC43	corona
1967	Human enterovirus D	picorna
1967	Piry virus	rhabdo
1967	Tacaiuma virus	bunya
1968	Human herpesvirus 2	herpes
1968	Marburg virus	filo
1968	Tataguine virus	bunya
1970	Everglades virus	toga
1970	Hepatitis B virus	hepadna
1970	Lassa virus	arena
1970	Punta Toro virus	bunya
1971	Aroa virus	flavi
1971	BK virus	polyoma
1971	Duvenhage virus	rhabdo
1971	JC virus	polyoma
1971	Vaccinia virus	pox
1972	Bovine papular stomatitis virus	pox
1972	Mokola virus	rhabdo
1972	Monkeypox virus	pox
1972	Norwalk virus	calici
1972	Ross River virus	toga
1973	Bangui virus	bunya
1973	Dugbe virus	bunya
1973	Hepatitis A virus	picorna
1973	Kotonkan virus	rhabdo
1973	Rotavirus A	reo
1973	Tamdy virus	bunya
1974	Getah virus	toga
1975	B19 virus	parvo
1975	Bhanja virus	bunya
1975	Human astrovirus	astro
1975	Lebombo virus	reo
1975	Shuni virus	bunya
1975	Thogoto virus	orthomyxo
1976	Orungo virus	reo
1976	Wanowrie virus	bunya
1977	Hepatitis delta virus	unassigned
1977	Sudan Ebola virus	filo
1977	Zaire Ebola virus	filo
1978	Hantaan virus	bunya
1978	Issyk-Kul virus	bunya
1980	Human T-lymphotropic virus 1	retro
1980	Puumala virus	bunya
1982	Human T-lymphotropic virus 2	retro
1982	Seoul virus	bunya
1983	Candiru virus	bunya
1983	Hepatitis E virus	unassigned
1983	Human adenovirus F	adeno
1983	Human immunodeficiency virus 1	retro
1984	Human torovirus	corona
1984	Rotavirus B	reo
1985	Borna disease virus	borna
1986	European bat lyssavirus 2	rhabdo
1986	Human herpesvirus 6	herpes
1986	Human immunodeficiency virus 2	retro
1986	Kasokero virus	bunya
1986	Kokobera virus	flavi
1986	Rotavirus C	reo
1987	Dhori virus	orthomyxo
1987	Sealpox virus	pox
1987	Suid herpesvirus 1	herpes
1988	Barmah Forest virus	toga
1988	Picobirnavirus	birna
1989	European bat lyssavirus 1	rhabdo
1989	Hepatitis C virus	flavi
1990	Banna virus	reo
1990	Gan Gan virus	bunya
1990	Reston Ebola virus	filo
1990	Semliki Forest virus	toga
1990	Trubanaman virus	bunya
1991	Guanarito virus	arena
1992	Dobrava-Belgrade virus	bunya
1993	Sin Nombre virus	bunya
1994	Hendra virus	paramyxo
1994	Human herpesvirus 7	herpes
1994	Human herpesvirus 8	herpes
1994	Sabia virus	arena
1995	Bayou virus	bunya
1995	Black Creek Canal virus	bunya
1995	Cote d'Ivoire Ebola virus	filo
1995	Hepatitis G virus	flavi
1995	New York virus	bunya
1996	Andes virus	bunya
1996	Australian bat lyssavirus	rhabdo
1996	Juquitiba virus	bunya
1996	Usutu virus	flavi
1997	Laguna Negra virus	bunya
1998	Menangle virus	paramyxo
1999	Nipah virus	paramyxo
1999	Torque teno virus	circo
2000	Whitewater Arroyo virus	arena
2001	Baboon cytomegalovirus	herpes
2001	Human metapneumovirus	paramyxo
2003	SARS coronavirus	corona
2004	Human coronavirus NL63	corona
2005	Human bocavirus	parvo
2005	Human coronavirus HKU1	corona
2005	Human T-lymphotropic virus 3	retro
2005	Human T-lymphotropic virus 4	retro

a Full details of sources available from authors on request.

## References

[bib1] Allander T, Andreasson K, Gupta S, Bjerkner A, Bogdanovic G, Petersson M.A.A, Dalianis T, Ramquist T, Andersson B (2007). Identification of a third human polyoma virus. J. Virol.

[bib2] Bebber D.P, Marriot F.H.C, Gaston K.J, Harris S.A, Scotland R.W (2007). Predicting unknown species numbers using discovery curves. Proc. R. Soc. B.

[bib3] Chua K.B (2007). A previously unknown reovirus of bat origin is associated with an acute respiratory disease in humans. Proc. Natl Acad. Sci. USA.

[bib4] Cleaveland S, Laurenson M.K, Taylor L.H (2001). Diseases of humans and their domestic mammals: pathogen characteristics, host range and the risk of emergence. Phil. Trans. R. Soc. B.

[bib5] Dove A.D.M, Cribb T.H (2006). Species accumulation curves and their applications in parasite ecology. Trends Parasitol.

[bib6] Gaynor A.M (2007). Identification of a novel polyomavirus from patients with acute respiratory tract infections. PLoS Pathog.

[bib7] Jones K.E, Patel N.G, Levy M.A, Storeygard A, Balk D, Gittleman J.L, Daszak P (2008). Global trends in emerging infectious diseases. Nature.

[bib8] King D.A, Peckham C, Waage J.K, Brownlie J, Woolhouse M.E.J (2006). Infectious diseases: preparing for the future. Science.

[bib9] May R.M (1988). How many species are there on earth?. Science.

[bib10] Morse S.S (1995). Factors in the emergence of infectious diseases. Emerg. Infect. Dis.

[bib11] Storch, G. A. 2007 Diagnostic virology. In *Fields virology* (eds B. N. Fields, D. M. Knipe & P. M. Howley), pp. 565–604, 5th edn. Philadelphia, PA: Lippincott, Williams & Wilkins.

[bib12] Taylor L.H, Latham S.M, Woolhouse M.E.J (2001). Risk factors for human disease emergence. Phil. Trans. R. Soc. B.

[bib13] Wolfe N.D, Dunavan C.P, Diamond J (2007). Origins of major human infectious diseases. Nature.

[bib14] Woolhouse M.E.J, Gaunt E (2007). Ecological origins of novel human pathogens. Crit. Rev. Microbiol.

[bib15] Woolhouse M.E.J, Gowtage-Sequeria S (2005). Host range and emerging and re-emerging pathogens. Emerg. Infect. Dis.

